# Effectiveness of mHealth Interventions for Improving eHealth Literacy Among Patients With Chronic Diseases: Meta-Analysis and Systematic Review

**DOI:** 10.2196/82004

**Published:** 2026-04-17

**Authors:** Xueqin Yang, Yingping Ma, Yueting Wang, Mengting Liu, Xingyu Liu, Xueping Jiao, Fanghong Yan, Yuxia Ma, Lin Han, Yanan Zhang

**Affiliations:** 1 School of Nursing, Lanzhou University, No. 28, Yanxi Road, Chengguan District, Lanzhou, Gansu Province, 730000, China, +86 0931-8556807; 2 Department of Neurosurgery, The Second Affiliated Hospital of Lanzhou University, Lan Zhou, Gansu Province, Gansu, China; 3 Department of Neurosurgery, Gansu Provincial People's Hospital, Lan Zhou, Gansu, China; 4 Gansu Provincial People's Hospital, Lan Zhou, Gansu, China

**Keywords:** eHealth literacy, chronic diseases, mHealth intervention, meta-analysis, mobile health

## Abstract

**Background:**

With the widespread use of the internet and mobile devices, eHealth literacy promotion is critical for medical equity. Mobile health (mHealth) serves as a pivotal tool for enhancing eHealth literacy by providing accessible, interactive platforms for health information engagement. However, the evidence regarding the effectiveness of mHealth interventions on eHealth literacy among patients with chronic diseases remains inconclusive.

**Objective:**

This study aimed to evaluate the effectiveness of mHealth interventions on eHealth literacy among patients with chronic diseases based on randomized controlled trials (RCTs) and summarize supportive evidence from quasi-experimental and qualitative studies.

**Methods:**

A comprehensive search strategy was developed, and 8 electronic databases were systematically searched for studies published up to February 12, 2026. Patients with chronic diseases were included based on predefined inclusion criteria. The Cochrane risk of bias 2 tool for RCTs and the ROBINS-I tool for quasi-experimental studies were used to assess the risk of bias. Given the anticipated substantial heterogeneity among the studies included, we used a random-effects model based on the Hartung-Knapp-Sidik-Jonkman method to pool effect sizes. A narrative and quantitative synthesis of the findings was provided where appropriate.

**Results:**

A total of 15 studies were included in this review, including 6 RCTs, 5 quasi-experimental studies, and 4 qualitative studies, involving a total of 2884 patients with chronic diseases. Meta-analyses of RCTs suggested that mHealth interventions could improve eHealth literacy, with a pooled mean effect size of standardized mean difference (SMD)=1. 19 (95% CI 0.14-2.23; *P=*.03; *I²*=97.75%; PI [prediction interval]=−2.68 to 5.05). Subgroup analyses by intervention targets showed that interventions on targets with specific disease produced larger mean effects (SMD=1.61; 95% CI 0.16-3.06; PI=−5.40 to 8.63), while interventions targeting the population with general chronic diseases produced smaller effects (SMD=0.36; 95% CI 0-0. 73; PI=−0. 21 to 0. 94). Analysis by intervention duration subgroup showed that the combined effect of studies with intervention duration <3 months was statistically significant (SMD=0.61; 95% CI 0.09-1.13; *I²*=88.04%; PI=−5.72 to 6.95); while the combined effect of studies with intervention duration ≥3 months was not statistically significant. Taking into account bias and the risk of GRADE (Grading of Recommendations, Assessment, Development, and Evaluation), the certainty of RCT evidence was moderate, and the certainty of quasi-experimental evidence was low.

**Conclusions:**

mHealth interventions could improve eHealth literacy among patients with chronic diseases on average. By using prediction intervals, this study reveals that the effectiveness of mHealth interventions is highly context-dependent and closely linked to implementation factors. Advancing beyond prior work, this study centers on eHealth literacy as a core outcome and integrates multiple types of evidence. Meanwhile, this finding emphasizes the need for evidence-based intervention programs and more rigorous implementation of intervention designs in future research.

## Introduction

Chronic diseases pose a threat to population health across the life course, particularly affecting older adults, and have emerged as a major challenge in global public health. According to the World Health Organization, chronic diseases, including cardiovascular diseases, cancers, chronic respiratory diseases, and diabetes, lead to approximately 41 million deaths globally each year, accounting for 74% of all-cause deaths [1], with type 2 diabetes mellitus, cardiovascular diseases, cancers, and respiratory diseases accounting for 80% of premature deaths [2]. Moreover, factors such as population aging, lifestyle changes such as smoking, physical inactivity, and environmental pollution increase the incidence of patients with chronic diseases [3]. To address the rising prevalence of patients with chronic diseases, effective strategies for managing chronic diseases are urgently needed.

eHealth literacy is an extension of health literacy, underpinned by digital literacy as its technical foundation. It refers to an individual’s comprehensive ability to use electronic resources to obtain, process, evaluate, and apply health information. eHealth literacy is defined as the ability of individuals to seek, find, understand, and appraise health information from electronic sources and apply the knowledge gained to address or solve a health problem [4], whose development relies upon the synergistic interplay of cognition, skills, motivation, and self-efficacy, rather than being a mere outcome variable. Studies have reported that enhanced eHealth literacy empowers patients to leverage online resources, telemedicine, and mobile health (mHealth) apps, thereby promoting treatment adherence and health outcomes [5]. Several studies showed eHealth literacy was insufficient among patients with chronic diseases. Zhenxiang et al [6] reported that only 5% of hospitalized patients with stroke in tertiary hospitals achieved adequate health literacy. Similarly, Sadeghi et al [7] reported that only 38.5% of patients with type 2 diabetes mellitus had adequate eHealth literacy, with 61.5% exhibiting low literacy. Moreover, studies reported that inadequate health literacy among patients with chronic diseases was associated with increased mortality and readmission rates [8,9], decreased quality of life [10], and increased disease-related health care costs [11]. Studies collectively underscore that inadequate health literacy impedes self-management and preventive care adherence in chronic disease populations [12,13]. Despite the potential of digital tools, many patients exhibit low eHealth literacy, limiting their capacity to effectively navigate and use these technologies. Consequently, improving eHealth literacy among patients with chronic diseases has become an urgent priority [14]. Improving eHealth literacy is key to achieving medical equity. It bridges the digital divide for vulnerable patients, ensures a fairer distribution of mHealth benefits, and reduces disparities in chronic disease outcomes.

mHealth is defined as “medical and public health practice supported by mobile devices, such as mobile phones, patient monitoring devices, personal digital assistants, and other wireless devices” [15]. Compared with traditional education, mHealth can offer multimodal learning materials (text, audio, video, and images), interactive functions (reminders, quizzes, and goal tracking), and repeated practice opportunities. The Lily model proposed by Norman posits that the abilities to obtain, communicate, and engage are core skills. And mHealth could promote eHealth literacy through convenient access to health care resources, facilitating doctor-patient communication and interaction, and enabling personalized feedback and monitoring [16,17]. The Integrative Model of eHealth Use theory posits that eHealth literacy serves as a crucial mediating variable for health outcomes, including behavioral and prognostic outcomes [18]. For instance, Qiu et al [19] research reported that mHealth apps could enhance the eHealth literacy and thereby improve the quality of life for older adult populations. Melholt et al [20] developed an interactive cardiac telerehabilitation tool within the dedicated online platform “Vital Heart,” and found that rehabilitation interventions delivered via text, video, and images significantly enhance eHealth literacy among patients with chronic diseases. However, He et al [21] conducted a pilot study in Hong Kong where trained university students provided home-based, personalized mHealth apps for older adult participants and found that eHealth literacy was not significantly improved, suggesting that mHealth might limit the effectiveness in enhancing electronic health literacy. Given the great advantages of mHealth interventions in enhancing eHealth literacy, it is urgently needed to explore their effectiveness on eHealth literacy among patients with chronic diseases.

Hence, despite the potential of mHealth tools to improve eHealth literacy and health outcomes, few studies have examined the effectiveness of mHealth interventions on enhancing eHealth literacy among patients with chronic diseases. Although many researchers have explored strategies to improve eHealth literacy among patients with chronic diseases, existing studies remain limited by heterogeneous methodologies, inconsistent outcomes, and a lack of theoretical grounding [22,23]. Hence, there is an urgent need for rigorous, standardized approaches to evaluate intervention effectiveness, with randomized controlled trials (RCTs) providing the primary basis for causal inference, and to optimize eHealth literacy interventions for patients with chronic diseases. Therefore, we conducted this study to evaluate the effectiveness of mHealth interventions on eHealth literacy among patients with chronic diseases based on RCT evidence, while synthesizing quasi-experimental and qualitative studies as supportive and contextual evidence. Our findings will provide evidence for designing targeted, scalable mHealth strategies.

## Methods

### Data Sources and Search Strategy

We performed this systematic review and meta-analysis in accordance with the PICOS (population, intervention, comparator, outcome, and study design) framework and PRISMA-S (Preferred Reporting Items for Systematic Reviews and Meta-Analyses–Search) guidelines [24] (Checklist 1). The study protocol was registered with PROSPERO (CRD42024622807). All databases were searched individually on their native platforms (no multidatabase platform searching was used). Two researchers (XY and YW) independently conducted a thorough search of all relevant studies published in the following databases: PubMed, Embase, Web of Science, Cochrane Library, WanFang Database, China National Knowledge Infrastructure (CNKI), CQVIP, and Chinese Biomedical Literature (CBM) from inception to February 2026 to ensure no recent studies had been published since the initial search. No study registries (eg, ClinicalTrials.gov and World Health Organization International Clinical Trials Registry Platform) were searched. No additional online resources or print sources (eg, conference proceedings and websites) were purposefully searched or browsed. Medical subject headings terms and free words were used in combination to search the relevant studies. No published search filters were used in any of the database searches. The search strategies were developed de novo by the authors based on the PICOS framework and were not adapted from prior reviews. To maximize sensitivity for the population concept, the chronic disease component was searched using both generic terms (eg, chronic disease or illness, noncommunicable disease, and multimorbidity or comorbidity) and comprehensive controlled vocabulary or keywords covering major chronic conditions (cardiovascular diseases, cancers or neoplasms, chronic respiratory diseases, diabetes, hypertension, stroke or cerebrovascular disease, chronic kidney disease, and chronic liver disease). In addition, the language of the studies was set as English and Chinese. The references of pertinent previous systematic reviews were also screened as supplementary sources. We did not contact authors, experts, or manufacturers to seek additional studies or data. No additional search methods beyond those described were used. Following the completion of the initial draft, a supplementary literature search was conducted in February 2026 to identify any newly published studies (as noted in Deviations from the Registered Protocol). No automatic email alerts were used. The search strategies were developed by 2 reviewers (XY and YW) and reviewed by a third reviewer (XL) for completeness and accuracy; however, no formal peer review of the search (eg, using PRESS [Peer Review of Electronic Search Strategies]) was conducted. The full search strategies were shown in Tables S1 to S8 (Multimedia Appendix 1).

### Eligibility Criteria

Eligibility criteria were set based on the PICOS framework:

1. Population: chronic disease has various definitions, which generally include the concepts that the condition is long-lasting, daily life is impacted, and ongoing management is required [25]. In our retrieval strategy, we used common chronic disease terminology, people with chronic illnesses (diabetes, high blood pressure, heart disease, lung disease, kidney disease, cancer, stroke, or any combination of these conditions that meet the diagnostic criteria for clinical diseases).
2. Interventions: mHealth-based interventions aimed at improving eHealth literacy in patients with chronic diseases. Eligible interventions included: mHealth apps (eg, disease management apps and medication reminders), SMS or text message–based health education, telehealth consultations via mobile devices, interactive mobile learning modules, and wearable device–integrated education programs. All interventions required primary delivery through mobile platforms (smartphones or tablets) with at least one eHealth literacy outcome measure.
3. Control: the study subjects in the control group received either no intervention at all or conventional interventions (eg, health education or general nursing practices).
4. Outcome: eHealth literacy score. The primary outcome measure of this study is the change in patients’ eHealth literacy scores, assessed using validated scales such as eHealth Literacy Scale (eHEALS). This indicator was selected as the core basis for evaluating effectiveness (primarily within RCTs). In line with best practice, meta-analyses were conducted separately by study design (like-for-like pooling): RCTs were pooled only with RCTs, and quasi-experimental studies were pooled only with quasi-experimental studies; different designs were not combined within the same meta-analysis. The primary outcome was the eHealth literacy score. This outcome was selected because eHealth literacy represents both a key prerequisite capability and a direct outcome for patients to effectively use mHealth and engage in self-management. Given the anticipated clinical and methodological diversity, we used a random-effects model for all quantitative syntheses. Because eHealth literacy represents both a key prerequisite capability and a direct outcome for patients to effectively use mHealth and engage in self-management. It serves as a fundamental and widely recognized surrogate measure for determining whether interventions are effective.
5. Research design: RCTs and quasi-experimental studies were included in this systematic review and meta-analysis. In the former, the experimental group receives mHealth intervention, while the latter group either receives no intervention or traditional interventions (mostly written or spoken health education materials). Nonexperimental trials, review or discussion papers, non-Chinese and non-English papers, studies with no full text available, and studies with inadequate data were excluded.

### Study Selection

A thorough screening procedure was conducted in compliance with PRISMA 2020 and 2 reviewers (XY and YW) screened the included studies independently. Among the initial 4057 records imported into EndNote X9 software, a total of 1142 duplicate publications were identified through a combination of EndNote software and manual verification. Subsequently, studies were excluded based on inclusion and exclusion criteria by reviewing titles and abstracts. Finally, full-text articles were reviewed for eligibility. Discussions or negotiations with a third reviewer (XL) were used to settle disagreements.

### Data Extraction and Quality Assessment

XY and YM extracted data from each study separately. Study authors, publication date, country, population (population and sample size), study design (description of numerical interventions, characteristics of intervention groups, and duration), theoretical framework, intervention site outcome indicators (measurement tools), and primary research outcome were extracted. The author team engaged in discussions and reexamined the original study in cases where there were disagreements over data extraction until an agreement was reached. The quality of evidence was assessed using the GRADE (Grading of Recommendations, Assessment, Development, and Evaluation) professional guidance development tool. Evidence quality was determined by evaluating five aspects: (1) risk of bias, (2) inconsistency, (3) indirectness, (4) imprecision, and (5) small-study effects [26].

### Risk of Bias Assessment

This study used the risk-of-bias assessment tools recommended by the Cochrane Collaboration, evaluating studies according to their specific design types. For RCTs, the Cochrane Risk of Bias 2 (RoB 2) tool was used [27]. This tool evaluates six domains: random sequence generation, allocation concealment, blinding of participants and personnel, blinding of outcome data, selective reporting, and other bias. Each domain is rated as “Low risk of bias,” “High risk of bias,” or “Unclear risk of bias.” Bias in nonrandomized studies (eg, quasi-experimental designs) was appraised using the ROBINS-I tool specifically designed for such intervention studies [28]. The 7 domains of bias addressed in the ROBINS-I assessment tool include bias due to confounding, bias arising from measurement of the exposure, bias in the selection of participants into the study, bias due to post-exposure interventions, bias due to missing data, bias in measurement of the outcome, and bias in selection of the reported result. Each domain is addressed using a series of signaling questions that aim to gather relevant information about the study and analysis being assessed. Most signaling questions offer five response options: “Yes,” “Probably yes,” “Probably no,” “No,” and “No information.” Responses of “Yes” and “Probably yes” are treated equivalently in risk-of-bias judgments, as are “No” and “Probably no.” The categories for risk of bias judgments are “No information,” “Low risk,” “Moderate risk,” “Serious risk,” and “Critical risk” of bias. All assessments were conducted independently by 2 reviewers (XY and YT). Disagreements were resolved through joint discussion or consultation with a third reviewer (XL).

### Small-Study Effects

As the funnel plot requires at least 10 original studies, this study used regression analysis with Egger test to assess small-study effects. The results indicated that the intercept term β₁ was not statistically significant (β₁=2.22, SE=1.83; *P*=.22), suggesting no significant small-study effects were detected in the current analysis, as shown in Table 1. For details on testing small-study effects in quasi-experimental studies, see Table S9 (Multimedia Appendix 1).

**Table 1.** Egger test for small-study effects in randomized controlled trial (n=6)^a^.

Terms	Values
β₁	2.22
SE	1.827
z	1.21
*P*	.22

a The regression-based Egger test was performed under a random-effects model (Hartung–Knapp–Sidik–Jonkman estimator). H₀: β₁=0 (no small-study effects). The non-significant *P* value suggests no evidence of small-study effects was detected in the present set of studies. However, it is important to note that the statistical power of the Egger test is limited when the number of included studies is small (k=6 in this analysis). Therefore, this result should be interpreted with caution, and the possibility of publication bias cannot be definitively ruled out.

### Data Synthesis and Statistical Analysis

Although most included studies used Norman’s eHealth Literacy Scale [29], some used other validated instruments to measure eHealth literacy. Given the variability in assessment tools, the standardized mean difference (SMD) and its 95% CI were calculated to enable pooling of continuous outcome data. In line with best practice, meta-analyses were conducted separately by study design (like-for-like pooling): RCTs were pooled only with RCTs, and quasi-experimental studies were pooled only with quasi-experimental studies; different designs were not combined within the same meta-analysis. Given anticipated clinical and methodological diversity, we used a random-effects model for all quantitative syntheses. The Hartung-Knapp-Sidik-Jonkman method was used to construct the random-effects model for effect estimation and pooling. This method is strongly recommended in meta-analyses as it enables more reliable uncertainty estimation while reducing the rate of false positives [30]. All analyses were conducted in Stata 17 using the meta suite with the Hartung–Knapp–Sidik–Jonkman method. To investigate potential sources of heterogeneity, we conducted pre-specified subgroup analyses based on target of intervention (specific chronic disease vs chronic disease in general) and intervention duration (≥3 mo vs <3 mo). Studies were sequentially excluded in sensitivity analyses. Egger tests were used to assess small-study effects for meta-analyses that involved more than 10 studies [31]. *P*<.05 was set as statistically significant.

### Prediction Interval Calculation

To quantify the real-world impact of heterogeneity, we computed the 95% prediction interval (PI) for the pooled overall estimate. Due to the limited number of included studies (k=6) and substantial between-study heterogeneity, we applied the confidence distribution method proposed by Nagashima et al [32], which is recommended for small-sample meta-analyses. PIs were presented for meta-analyses with a sufficient number of studies (≥3). When the number of studies in a subgroup was small, PI estimates were interpreted cautiously.

### Deviations From the Registered Protocol

During the implementation of this study, several adjustments were made to the protocol pre-registered on the PROSPERO platform (CRD42024622807). Following the completion of the initial draft, a supplementary literature search was conducted in February 2026, resulting in the inclusion of 15 studies [16,20,33-45] that met the eligibility criteria. Given the limited number of included studies, small-study effects were assessed using the Egger test instead of the initially planned funnel plot analysis. For data synthesis, the Hartung-Knapp-Sidik-Jonkman random-effects model was used to pool effect sizes. The literature search was restricted to publications in Chinese and English. All adjustments were documented throughout the research process and were deemed unlikely to substantially affect the validity of the study conclusions.

## Results

### Selection of the Studies

**Figure 1.**
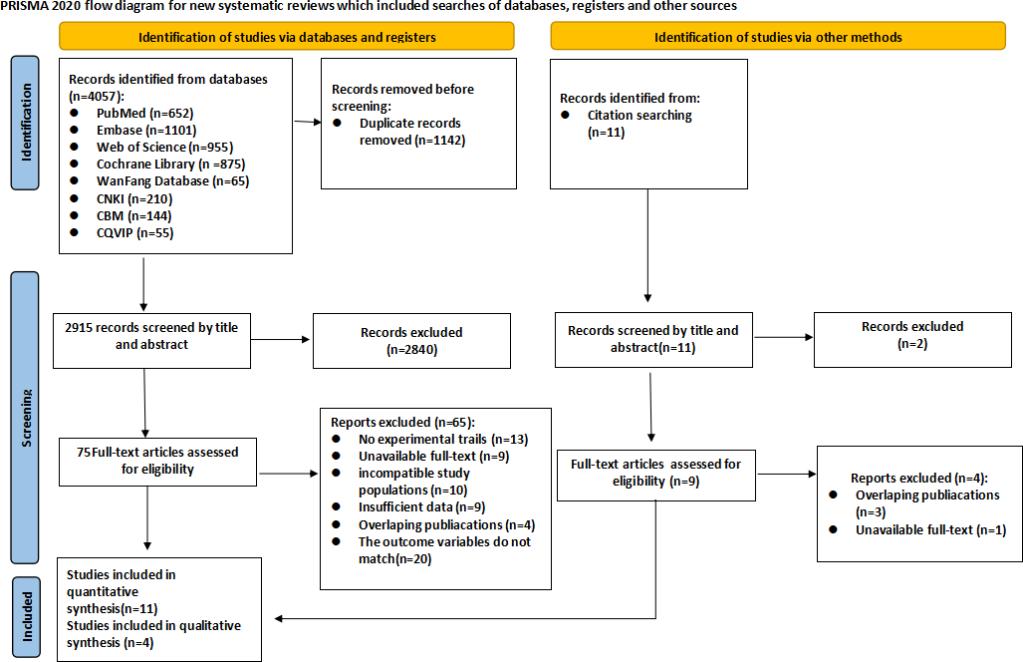
PRISMA (Preferred Reporting Items for Systematic Reviews and Meta-Analyses) flow diagram of the literature selection process for the systematic review on the effectiveness of mobile health interventions on eHealth literacy among patients with chronic diseases (studies published between 2016 and 2026).

The screening procedure for the subsequent subphases (title or abstract screening, full text assessment) was strictly followed by the PRISMA framework to ensure transparency and reproducibility. Initially, 4057 articles were searched, including PubMed (n=652), Embase (n=110 1), Web of Science (n=955), Cochrane Library (n=875), the Wan Fang Database (n=65), the CBM (n=144), the CQVIP (n=55), and the CNKI (n=210). Using EndNote software, 1142 duplicate references were excluded. The remaining 2915 papers were screened by title and abstract. A total of 2840 papers were excluded due to noncompliance with inclusion criteria or irrelevance to the research topic. After full-text review of the remaining 84 studies, 69 were excluded for reasons detailed in the supplementary file. (Multimedia Appendix 2). The final analysis incorporated 15 eligible studies [16,20,33-45], comprising 6 RCTs [33,38-40,44,45] for the primary quantitative synthesis, 5 quasi-experimental studies [34-37,43] for supplementary quantitative synthesis, and 4 qualitative studies [16,20,41,42] were included and synthesized narratively. Figure 1 shows the comprehensive flowchart of the literature search process.

### Study Characteristics

15 clinical studies [16,20,33-45] were reviewed and 2884 participants with various chronic conditions were included. Geographically, the studies were predominantly conducted in China (n=7) [33-39], Australia (n=2) [40,41], Canada (n=1) [42], Denmark (n=2) [16,20], South Korea (n=1) [43], and the United States [44,45]. All studies were published between 2016 and 2026. Two of the papers were published in Chinese, while thirteen were published in English. Regarding intervention formats, the 15 included studies predominantly used blended online-offline models (n=8) [33-36,38-40,45], followed by purely online interventions (n=4) [20,41,42,44], and face-to-face interventions (n=3) [16,37,43]. Regarding intervention content, the primary focus encompassed eHealth literacy skills training (n=6) [33,34,36-39], disease self-management and monitoring (n=5) [16,20,35,39,40], health knowledge dissemination (n=4) [16,20,35,41], and social interaction and support (n=3) [16,39,44]. Regarding intervention methodologies, approximately half of the studies were designed based on explicit theoretical frameworks (n=9) [16,33,35,37-39,42-44], with widespread adoption of personalized feedback (n=4) [16,35,40,43], interactive learning (n=5) [16,33,35,36,43], and multimedia instruction (n=5) [16,20,36,37,44], to enhance patient engagement and learning outcomes. The key features of the 15 studies are presented in Table 2. A summary of intervention implementation is provided in Table S10 (Multimedia Appendix 1).

**Table 2.** Characteristics of included randomized controlled trials and quasi-experimental studies on mobile health (mHealth) interventions for improving eHealth literacy in patients with chronic diseases (n=15).

Study design	Author (year)	Country	Total (intervention/control)	Men/ women	Age (years), mean (SD)	Intervention type	Intervention content	Intervention population	Theory	Duration of intervention
RCT^a^	Yu [33] (2022)	China	58 (29/29)	19/39	51.03 (7.25)	Theory-based digital program (TMeHL)	Four-stage eHealth literacy training (positioning, communication, evaluation, and application).	Young or middle-aged stroke inpatients (18‐59 years)	Transactional Model of eHealth Literacy	12 weeks
RCT	Nahm et al [44] (2019)	America	272 (138/134)	81/191	70 (8.5)	Online education program (T-PeP)	Structured learning modules + moderated discussions + virtual library.	Community-dwelling older adults (50+ years) with chronic conditions	Self-Efficacy Theory	3 weeks
QED^b^	Hu [34] (2024)	China	73 (38/35)	48/25	—	Blended online/offline training	Health information searching/evaluation/application practice.	Older patients with stroke (≥60 years) with internet experience	—	1‐2 weeks
QED	Jiang [35] (2024)	China	60 (30/30)	31/29	—	WeChat-based mini-program	Health education + blood pressure monitoring + offline event coordination.	Hypertensive adults (18+ years) with smartphone proficiency	Health consciousness theory	3 months
QED	Guo et al [36] (2023)	China	132（96/36）	93/39	41.22 (8.42)	Mobile eHealth education	Diabetes app navigation + reliable website identification.	Adults with type 2 diabetes (20‐65 years)	—	3 months
RCT	Parker et al [40] (2022)	Australia	215（120/95）	123/92	58.9 (8.8)	Nurse-led multifaceted intervention	Health checks + lifestyle app (mysnapp) + telephone coaching.	Adults with overweight or obesity (40‐74 years) with low health literacy	—	6 months
QED	Chiu et al [37] (2016)	Taiwan, China	39（39/20）	14/25	69.5 (6.9)	Touchscreen device training	Health/entertainment/social app tutorials.	Older adults (50+ years) with limited internet experience	Technology Acceptance Model	8 weeks
RCT	Redfern et al [41] (2020)	Australia	934 (486/448)	716/218	67.6 (8.1)	EHR^c^-integrated web application	CVD^d^ risk assessment + goal-setting + social features.	Adults at high CVD risk (≥18 years)	—	Follow-up 12 months
RCT	Kastner et al [42] (2021)	Canada	440（220/220)	—	—	Web-based self-management tool (Keep Well)	Personalized lifestyle advice + multicondition tracking.	Older adults (≥65 years) with multimorbidity	Knowledge-to-Action (KTA) model	6 months
QED	Melholt et al [20] (2017)	Denmark	109 (49/49)	40/9	60.64 (10.75)	Cardiac telerehabilitation portal	Multimedia disease management resources + self-monitoring.	Adult patients with cardiac issues (≥18 years) postsurgery or with heart failure	—	3 month
RCT	Cheng et al [38] (2024)	Taiwan, China	92 (46/46)	33/59	62.38 (12.9)	eHealth care experiential learning program	experiential learning program based on eHealth literacy framework (eHLF) and experiential learning theory (ELT), covering use of eHealth tools for diabetes self‑management.	Patients with type 2 diabetes, aged ≥20 years, owning mobile phone or tablet with internet	eHLF and ELT	3 months
QED	Son et al [43] (2023)	South Korea	100 (50/50)	83/17	58.78 (8.83)	Interactive text message‑based mHealth intervention (WithUs)	Weekly goal setting + interactive text messaging (chatbot) for heart failure self‑care.	Patients with heart failure aged ≥40 years, owning Android smartphone	Information‑Motivation‑Behavioral skills model (IMB)	6 months
RCT	Spindler et al [16] (2022)	Denmark	137 (67/70)	105/32	61.5 (11.1)	Telerehabilitation program (HeartPortal)	Digital toolbox with educational materials, self‑tracking (blood pressure, weight, and steps), communication with health professionals, goal setting, and patient‑reported outcomes monitoring.	Patients with heart failure (NYHA I‑IV), aged ≥18 years	Self‑Determination Theory (SDT)	12 months
RCT	Gao et al [39] (2026)	China	130 (65/65)	108/22	73.92 (5.14)	Theory-guided eHealth literacy program (SRL-SEe PR)^e^	4-stage eHealth literacy course Self-learning via manuals/QR codes+ reflective journals+ remote WeChat support.	Older adults (≥65 years) with COPD^f^	Self-Efficacy theory	8 weeks
RCT	Lyles et al [45] (2019)	United States	93 (44/49)	45/48	54.3 (13)	Portal training program	In-person: research assistant–guided access to 11 video tutorials on patient portal use. Take-home: handout with link to self-access same 11 videos.	Vulnerable English-speaking adults (≥18 years) with chronic diseases and an email address	—	One-time session

a RCT: randomized controlled trial.

b QED: quasi-experiment design.

c EHR: electronic health record.

d CVD: cardiovascular disease.

e SRL-SEe PR: self-regulated learning model and self-efficacy theory.

f COPD: chronic obstructive pulmonary disease

### Risk of Bias Assessment

**Figure 2.**
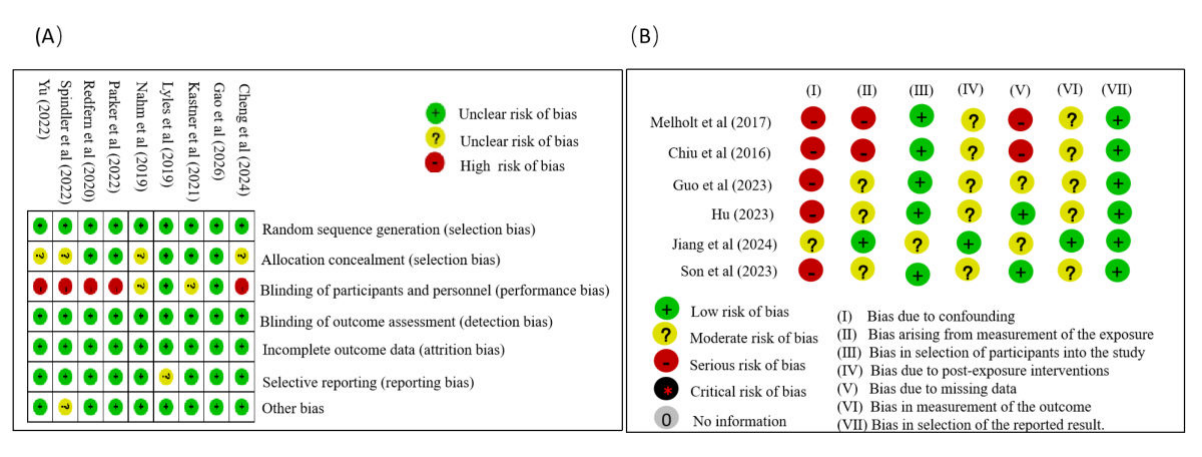
Summary of risk of bias assessment for included studies. (A) Risk of bias in randomized controlled trials (assessed using the Cochrane RoB 2 tool). (B) Risk of bias in non-randomized controlled trials (assessed using the ROBINS-I tool) [16,20,33-45].

A total of 15 studies [16,20,33-45] were included in this review. Of these, 11 studies contributed quantitative data to the meta-analyses (the primary meta-analysis pooled 6 RCTs [33,38-40,44,45], and the supplementary meta-analysis pooled 5 quasi-experimental studies [34-37,43], while the remaining 4 qualitative studies [16,20,41,42] were synthesized narratively. Importantly, risk-of-bias assessments were conducted for all included quantitative studies, regardless of whether they were ultimately pooled in the meta-analyses. Specifically, we used the Cochrane risk of bias assessment tool to evaluate the methodological quality of the 9 included RCTs [16,33,38-42,44,45,] (Figure 2A). Five studies [16,33,38,40,41] were judged to be at high risk of bias due to deviations from intended interventions or implementation issues. Four studies [16,33,38,44] had some concerns related to the randomization processor selection-bias–related information. Two studies [16,45] presented an unclear risk of reporting bias. Overall, the primary limitations of the RCT evidence centered on inadequate blinding and implementation, whereas other domains were relatively consistent, leading to an acceptable overall risk-of-bias profile. For the 6 quasi-experimental studies [20,34-37,43] included, we used the ROBINS-I tool for bias risk assessment (Figure 2B). The assessment revealed that 5 studies [20,34,35,37,43] exhibited a core issue of serious risk of confounding bias, and 2 studies [20,37] presented a high risk of bias due to missing data.

### Certainty of Evidence

The initial quality of evidence in this study was rated as high due to the inclusion of RCTs. However, the certainty of evidence was downgraded by one level owing to substantial heterogeneity between studies that could not be fully explained. Consequently, the overall quality of evidence supporting the conclusion that mHealth interventions can improve eHealth literacy among patients with chronic diseases is moderate**.** The GRADE evidence level for the pooled estimate from quasi-experimental studies was, as expected, rated as low due to the very serious risk of confounding bias inherent in their design. (Multimedia Appendix 3.)

### Overall Effectiveness of RCTs mHealth Interventions on eHealth Literacy for Patients With Chronic Diseases

**Figure 3.**
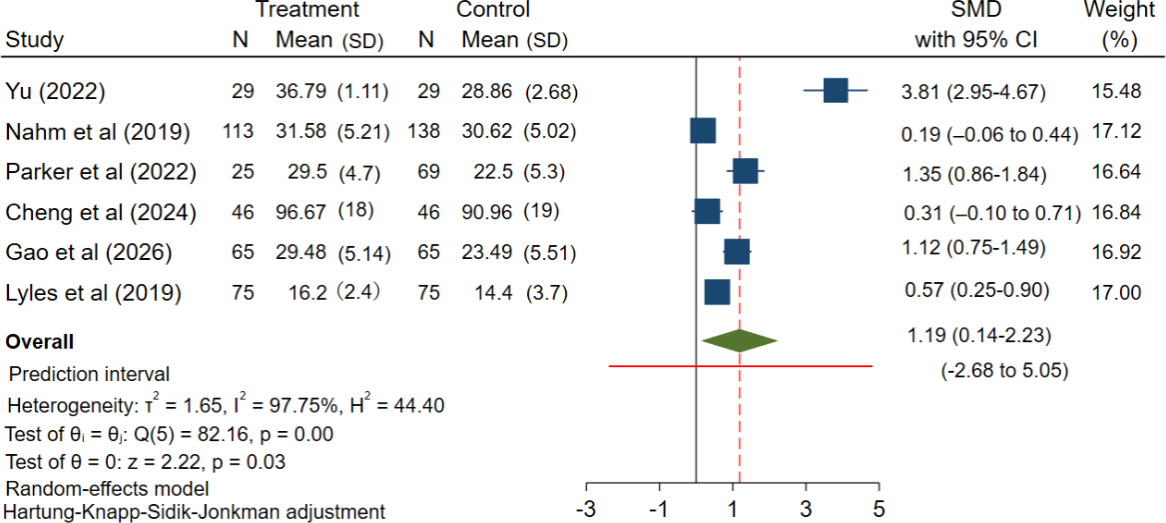
Forest plot of the meta-analysis of randomized controlled trials: effect of mHealth interventions on eHealth literacy in patients with chronic diseases. mHealth: mobile health; SMD: standardized mean difference [33,38-40,44,45].

**Figure 4.**
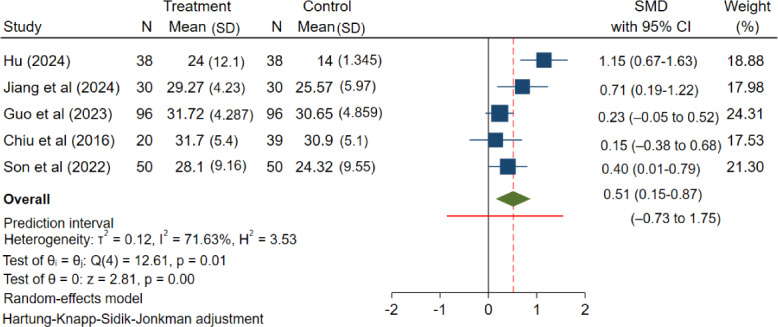
Forest plot of the meta-analysis of quasi-experimental studies: effect of mHealth interventions on eHealth literacy in patients with chronic diseases. mHealth: mobile health; SMD: standardized mean difference [34-37,43].

The effectiveness of eHealth literacy interventions in patients with chronic diseases was systematically assessed in this review, with effectiveness primarily assessed using RCT evidence. As explained below, specific data could be found in Table S11 (Multimedia Appendix 1). Quantitative syntheses followed a like-for-like approach by design: the primary meta-analysis pooled RCTs only (Figure 3 [33,38-40,44,45]), and a separate supplementary meta-analysis pooled quasi-experimental studies only (Figure 4 [34-37,43]).

### Primary Analysis: RCTs

The meta-analysis of 6 RCTs [33,38-40,44,45] showed that mHealth interventions can enhance eHealth literacy among patients with chronic diseases. The pooled average effect was positive (SMD=1.19; 95% CI 0.14-2.23; *P*=.03; Figure 3). The 95% CI quantifies uncertainty around this pooled average effect. Between-study heterogeneity was substantial; while *I²* is commonly reported (*I²*=97.75%, *P*<.001), it has limited pragmatic utility for understanding how much true effects vary across settings. Therefore, to describe the expected distribution of true effects across different populations and settings, we calculated the 95% PI, which was extremely wide (−2.68 to 5.05; Figure 3). Thus, although the average effect is beneficial, a future implementation could plausibly observe little or no benefit or even harm, or conversely, a very large benefit, depending on context and implementation. This interpretation is consistent with the risk-of-bias assessment (several trials had limitations related to blinding and implementation) and with the GRADE rating of moderate certainty for the RCT evidence, downgraded for unexplained inconsistency.

### Supplementary Analysis: Quasi-Experimental Studies

#### Overview

A separate meta-analysis of the 5 quasi-experimental studies [34-37,43] also indicated a positive pooled average effect (SMD=0.51; 95% CI 0.15 - 0.87; *P*<.001; Figure 4). Between-study heterogeneity was notable; although *I²* was moderate to high (*I²*=71.63%), the 95% PI was also broad (−0.73 to 1.75), indicating that true effects may range from little or no benefit to meaningful benefit across settings. Given the serious risk of bias (primarily confounding) and low-grade certainty associated with these studies, these results should be considered supportive but less robust than the primary RCT findings.

#### Analysis of Subgroups

We conducted subgroup analyses based on prespecified subgroups (including intervention targets and intervention duration), as detailed below, to more specifically explore potential effect modifiers in the RCT-based estimates of intervention effectiveness on eHealth literacy.

#### The Effect of mHealth Intervention on eHealth Literacy Varied by Intervention Targets

**Figure 5.**
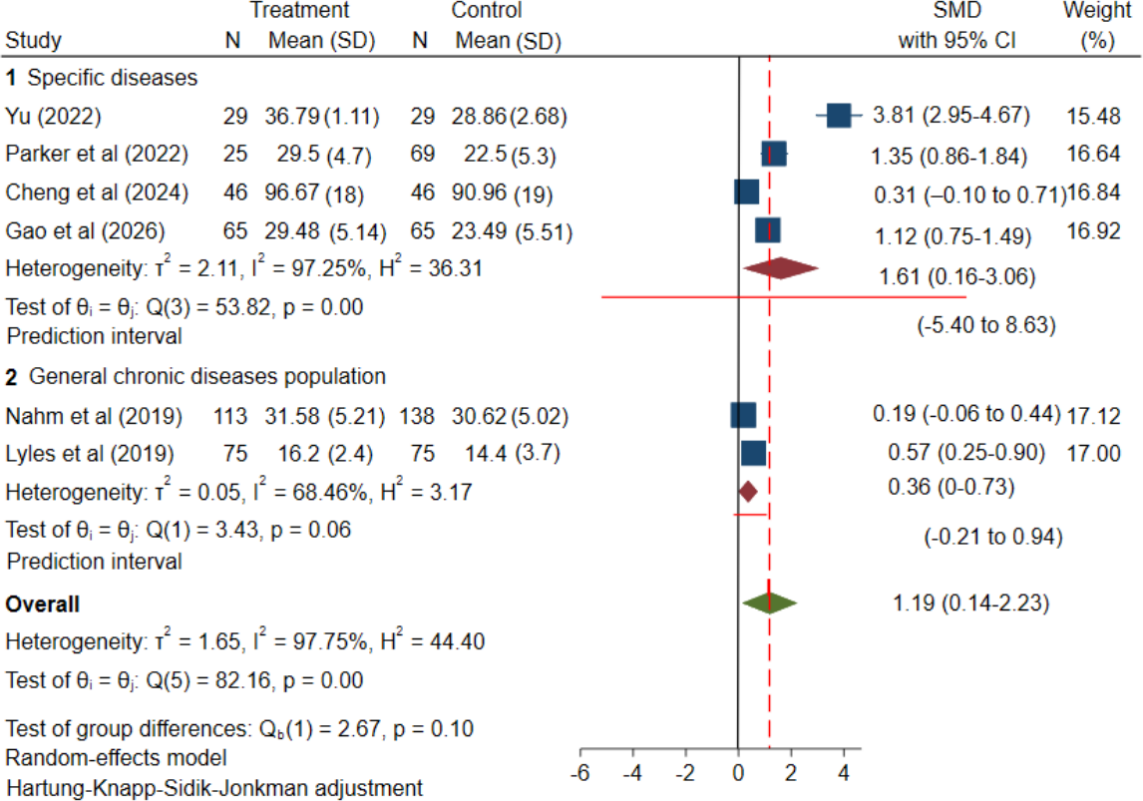
Subgroup analysis by intervention target: effect of mHealth interventions on eHealth literacy in patients with chronic diseases; SMD: standardized mean difference (Group 1: interventions targeting a specific disease [33,38-40]; Group 2: interventions targeting general chronic diseases population [44,45]).

After grouping by intervention target, specific disease interventions yielded a positive and substantial pooled average effect (SMD=1.61, 95% CI 0.16‐3.06; Figure 5). However, the 95% PI was very wide (PI −5.40 to 8.63), indicating that true effects may vary considerably across implementation contexts, ranging from apparent ineffectiveness or even negative outcomes to highly significant benefits. In contrast, interventions targeting the general chronic disease population yielded smaller average effects (SMD=0.36, 95% CI 0.00‐0.73; Figure 5 [33,38-40,44,45]), with a PI of −0.21 to 0.94. This implies that in certain contexts, the intervention may approach ineffectiveness, while in others, there remains a modest possibility of achieving some degree of positive effect. For completeness, conventional *I²* statistics are reported in Figure 5 (specific disease: *I²*=97.25%; general chronic disease population: *I²*=68.46%), but the PI provides the more clinically interpretable description of between-setting variability. The test for between-group differences failed to reach statistical significance (*P*=.10). Consequently, this grouping outcome should be regarded more as a suggestive trend than as a definitive effect modifier.

#### The Effect of mHealth Intervention on eHealth Literacy Varied by Duration of Intervention

**Figure 6.**
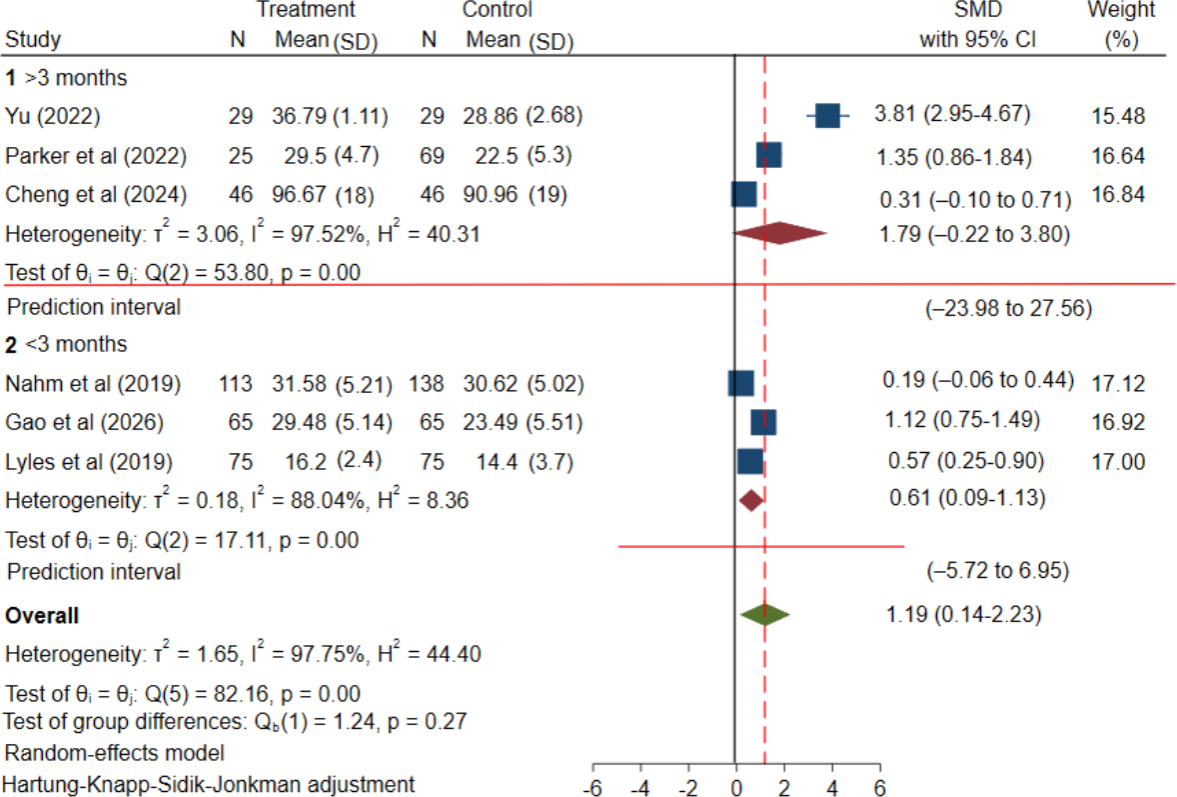
Subgroup analysis by intervention duration: effect of mHealth interventions on eHealth literacy in patients with chronic diseases; SMD: standardized mean difference (Group 1: intervention duration ≥3 months [33,38,40]; Group 2: intervention duration <3 months [39,44,45]).

After grouping by intervention duration, results indicated that the mean effect in the ≥3-month group was positive, yet its 95% CI encompassed zero (SMD=1.79, 95% CI −0.22 to 3.80; Figure 6 [33,38-40,44,45]). The corresponding PI was extremely wide (95% PI −23.98 to 27.56), suggesting that true effects may vary dramatically across studies and implementation contexts. In contrast, the group with an intervention duration of <3 months also exhibited a positive mean effect (SMD=0.61, 95% CI 0.09‐1.13; Figure 6) with a broad PI (PI −5.72 to 6.95). Although conventional *I²* values were reported in Figure 6 (≥3 mo: *I²*=97.52%;<3 mo: *I²*=88.04%), the PIs better quantify the likely range of effects in new settings. This suggests that short-term interventions may yield benefits in some contexts, yet the possibility of near-ineffectiveness or even negative outcomes persists.

#### Sensitivity Analyses

**Figure 7.**
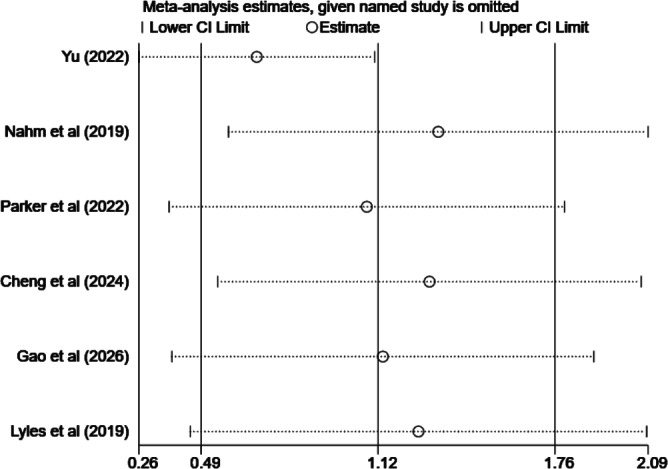
Leave-one-out sensitivity analysis of the pooled effect size for the effect of mHealth interventions on eHealth literacy in patients with chronic diseases [33,38-40,44,45].

The leave-one-out sensitivity analysis (Figure 7) demonstrated that no single study was solely responsible for the overall positive pooled effect. The point estimate remained positive and all CIs overlapped after the sequential exclusion of each study, indicating that the overall conclusion of an average positive effect is relatively robust to the influence of any one study.

## Discussion

### Principal Findings

This review aimed to understand whether mHealth tools could improve eHealth literacy among patients with chronic diseases. We used the results of RCTs as primary evidence. A combined analysis of 6 RCTs showed that the intervention had a positive effect on average. This study is consistent with previous research findings [46,47]. However, there are large differences between the studies. The 95% PI was wide. The wide pPI suggests that the effectiveness of the same intervention may differ substantially across implementation contexts, with true effects potentially ranging from negligible to clinically meaningful. Due to this large uncertainty and some problems with the study (such as the lack of blinding), we rated the overall quality of evidence from RCTs as moderate using the GRADE system [48,49]. We also synthesized 5 quasi-experimental studies, and the results of the quasi-experiment supported the results of RCT with large PI.

### Effectiveness of mHealth Interventions

The findings indicated that mHealth interventions are effective in enhancing eHealth literacy among patients with chronic diseases. The effect was more robust in RCTs, while quasi-experimental studies showed more modest effects accompanied by higher uncertainty. This result is generally consistent with previous reviews, which have shown that mHealth interventions are generally beneficial for health literacy-related outcomes, but the effects vary widely [50]. The observed differences may stem from heterogeneity in patient characteristics, intervention design, and outcome measures [51]. Lyles et al [45], in a study of underserved populations, found that portal use among individuals with limited health literacy was substantially lower than among those with adequate health literacy. Nahm et al [44] reported more favorable outcomes in older adults with higher educational attainment, suggesting that socioeconomic status and access to resources are critical determinants of intervention effectiveness. Intervention intensity and theoretical grounding also appear to be pivotal [52,53]. Gao et al [39] demonstrated that an intervention based on the self-regulated learning model was more effective than one relying on a single theoretical framework. Cheng et al [38] showed that experiential learning significantly improved eHealth literacy. By contrast, the findings of Parker et al [40] did not achieve meaningful improvements in clinical endpoints, likely owing to insufficient intervention intensity and limited tailoring. Future research should prioritize the development of stratified, adaptive, and theory-informed multimodal interventions [54]. In particular, greater attention should be directed toward designing and validating more personalized and supportive strategies for vulnerable populations with limited health literacy and inadequate social resources [55,56].

### Intervention Targets

This study conducted subgroup analyses by intervention target. The results showed that interventions designed for one specific disease (eg, diabetes alone or heart failure alone) appeared to work better than interventions designed for patients with all chronic conditions. This finding is consistent with previous research [57]. Interventions on patients with specific diseases showed greater effects on average. mHealth intervention programs targeting the general chronic disease population have smaller average effects, and their PIs suggest that they may not always be beneficial. When an intervention focuses on a specific disease, its content, tasks, and feedback closely match what patients need to do every day [58]. This may make the tool more useful to patients and keep them engaged [59]. In the field of mHealth, higher engagement generally leads to better outcomes [60,61]. For example, apps that help manage diabetes or blood pressure often include self-management tasks and provide direct feedback, which may explain why they work so well [62]. In contrast, trying to cover multiple conditions can make it difficult to provide in-depth, personalized support for each condition [51]. Recent research on tools for patients with multiple conditions showed that combining several disease-specific tools could feel burdensome and did not always provide useful, actionable recommendations [63,64]. Therefore, it may be wiser to start with small, sophisticated tools for a single disease and consider expanding to more diseases later.

### Duration of Intervention

We further compared studies with an intervention duration of less than 3 months versus an intervention duration of more than 3 months. Subgroup analysis showed a statistically significant combined effect of the less than 3-month intervention, which is generally consistent with the findings of Sevda and Mechelle [65,66]. However, the PI remained notably wide, suggesting considerable uncertainty in the effectiveness of the intervention when applied across diverse implementation contexts. In contrast, the subgroup with intervention for more than 3 months had larger point estimates, but its combined effect did not reach statistical significance, and the PI was extremely wide. Given that the test for subgroup differences did not reach statistical significance, it is unwarranted to conclude that long-term interventions are inherently less effective than short-term ones. A more plausible interpretation is that the effectiveness of longer-duration mHealth interventions may be more susceptible to contextual factors and the fidelity of implementation processes [67]. The possible reason is that the benefits of mHealth interventions depend not only on the duration of the intervention itself but also on whether patient engagement can be sustained over time [68]. Existing evidence suggests that user engagement in mHealth interventions often declines as time progresses, while higher levels of engagement are generally associated with better adherence and improved self-management outcomes [52]. For longer-duration interventions, factors such as treatment burden, reduced interaction, or implementation barriers may weaken sustained patient participation [69]. Therefore, future research should not only focus on extending the duration of interventions but also prioritize optimizing intervention content, feedback frequency, personalized support, and engagement strategies to enhance long-term adherence and participation, thereby maximizing the long-term effectiveness of mHealth interventions.

### Limitations

Four major limitations of this study should be mentioned. First, the restriction to English and Chinese language publications may introduce selection bias and limit the cross-cultural applicability of our findings. Second, there was considerable methodological heterogeneity among the studies included, particularly in terms of intervention protocols and outcome measures. Third, this study acknowledges certain limitations in the current analysis, primarily reflected in the relatively small sample size. Fourth, this study used eHealth literacy scores as the primary indicator of intervention effectiveness. While this metric directly reflects improvements in digital health capabilities, it struggles to comprehensively capture the intervention’s overall benefits regarding clinical symptoms, quality of life, and health care costs. Future research should adopt multidimensional indicators to conduct more comprehensive assessments.

### Conclusions

Overall, current evidence suggests that mHealth interventions can improve eHealth literacy among patients with chronic diseases on average. However, the wide PIs indicate substantial between-study variability, suggesting that intervention effects are highly context-dependent and closely related to implementation factors. By focusing on eHealth literacy as a core capability outcome and integrating evidence from RCTs, quasi-experimental studies, and qualitative research, this review extends previous work in the field. These findings highlight the need for more rigorously designed and better-implemented mHealth interventions in future research.

## Supplementary material

10.2196/82004 Multimedia Appendix 1 Supplementary data.

10.2196/82004 Multimedia Appendix 2 Studies excluded during full-text screening and reasons.

10.2196/82004 Multimedia Appendix 3 GRADE (Grading of Recommendations, Assessment, Development, and Evaluations) for evidence.

10.2196/82004 Checklist 1 PRISMA-S checklist.

10.2196/82004 Checklist 2 PRISMA 2020 checklist.

## Abbreviations

CBM: Chinese Biomedical Literature
CNKI: China National Knowledge Infrastructure
eHEALS: eHealth Literacy Scale
GRADE: Grading of Recommendations, Assessment, Development, and Evaluation
mHealth: mobile health
PI: prediction interval
PICOS: population, intervention, comparator, outcome, and study design
PRISMA-S: Preferred Reporting Items for Systematic Reviews and Meta-Analyses–Search
RCT: randomized controlled trial
SMD: standardized mean difference
