# Supplementary material for: Effectiveness of mHealth Interventions for Improving eHealth Literacy Among Patients With Chronic Diseases: Meta-Analysis and Systematic Review
Source: J Med Internet Res. 2026 Apr 17;28:e82004. doi: 10.2196/82004 (PMC13089675; doi:10.2196/82004)
Supplement: Multimedia Appendix 1 [file jmir-v28-e82004-s001.docx]

**Supplementary Table 1. Search strategy for PubMed on mHealth interventions and eHealth literacy in patients with chronic diseases (search date up to February 12, 2026).**

| **Number** | **Search terms** |
| --- | --- |
| #1 | Chronic Disease[Mesh] |
| #2 | Cardiovascular Diseases[Mesh] OR Heart Diseases[Mesh] OR Coronary Disease[Mesh] |
| #3 | Neoplasms[Mesh] |
| #4 | Respiratory Tract Diseases[Mesh] OR Lung Diseases[Mesh] OR Pulmonary Disease, Chronic Obstructive[Mesh] OR Asthma[Mesh] |
| #5 | Diabetes Mellitus[Mesh] |
| #6 | Hypertension[Mesh] |
| #7 | Stroke[Mesh] OR Cerebrovascular Disorders[Mesh] |
| #8 | Kidney Diseases[Mesh] OR Renal Insufficiency, Chronic[Mesh] OR Chronic Kidney Disease[Mesh] OR Liver Diseases[Mesh] |
| #9 | #1 OR #2 OR #3 OR #4 OR #5 OR #6 OR #7 OR #8 |
| #10 | ("chronic disease*"[tiab] OR "chronic illness*"[tiab] OR "noncommunicable disease*"[tiab] OR ncd*[tiab] OR multimorbid*[tiab] OR comorbid*[tiab] OR "cardiovascular disease*"[tiab] OR "heart disease*"[tiab] OR coronary[tiab] OR "coronary artery disease"[tiab] OR "myocardial infarct*"[tiab] OR atherosclero*[tiab] OR "heart failure"[tiab] OR hypertens*[tiab] OR diabetes[tiab] OR diabetic*[tiab] OR "type 2 diabetes"[tiab] OR t2dm[tiab] OR "type 1 diabetes"[tiab] OR neoplasm*[tiab] OR cancer*[tiab] OR tumor*[tiab] OR tumour*[tiab] OR malignan*[tiab] OR carcinoma*[tiab] OR "chronic respiratory disease*"[tiab] OR copd[tiab] OR "chronic obstructive pulmonary disease"[tiab] OR emphysema[tiab] OR "chronic bronchitis"[tiab] OR asthma[tiab] OR stroke[tiab] OR cerebrovascular[tiab] OR "brain attack"[tiab] OR "chronic kidney disease"[tiab] OR ckd[tiab] OR renal[tiab] OR nephropath*[tiab] OR "kidney disease*"[tiab] OR "renal insufficien*"[tiab] OR "liver disease*"[tiab] OR hepatic[tiab] OR cirrhosis[tiab]) |
| #11 | #9 OR #10 |
| #12 | ("eHealth literacy"[tiab] OR "e-health literacy"[tiab] OR ehealth literacy[tiab] OR "digital health literacy"[tiab] OR "digital literacy"[tiab] OR "computer literacy"[tiab] OR "internet literacy"[tiab] OR "telehealth literacy"[tiab] OR "mhealth literacy"[tiab] OR "mobile health literacy"[tiab]) |
| #13 | (interven*[tiab] OR trial*[tiab] OR program*[tiab] OR training[tiab] OR education[tiab] OR random*[tiab] OR "quasi-experiment*"[tiab] OR controlled[tiab]) |
| #14 | #11 AND #12 AND #13 |

**Supplementary Table 2. Search strategy for Embase on mHealth interventions and eHealth literacy in patients with chronic diseases (search date up to February 12, 2026)..**

| **Number** | **Search terms** |
| --- | --- |
| #1 | 'chronic disease'/exp OR 'cardiovascular disease'/exp OR 'heart disease'/exp OR 'coronary artery disease'/exp OR 'diabetes mellitus'/exp OR 'hypertension'/exp OR 'stroke'/exp OR 'cerebrovascular disease'/exp OR 'chronic obstructive lung disease'/exp OR 'asthma'/exp OR 'kidney disease'/exp OR 'chronic kidney disease'/exp OR 'renal insufficiency'/exp OR 'liver disease'/exp OR 'neoplasm'/exp |
| #2 | 'chronic disease*':ti,ab,kw OR 'chronic illness*':ti,ab,kw OR 'noncommunicable disease*':ti,ab,kw OR ncd*:ti,ab,kw OR multimorbid*:ti,ab,kw OR comorbid*:ti,ab,kw OR 'cardiovascular disease*':ti,ab,kw OR 'heart disease*':ti,ab,kw OR coronary:ti,ab,kw OR 'coronary artery disease':ti,ab,kw OR 'myocardial infarct*':ti,ab,kw OR atherosclero*:ti,ab,kw OR 'heart failure':ti,ab,kw OR hypertens*:ti,ab,kw OR diabetes:ti,ab,kw OR diabetic*:ti,ab,kw OR 'type 2 diabetes':ti,ab,kw OR t2dm:ti,ab,kw OR 'type 1 diabetes':ti,ab,kw OR neoplasm*:ti,ab,kw OR cancer*:ti,ab,kw OR tumor*:ti,ab,kw OR tumour*:ti,ab,kw OR malignan*:ti,ab,kw OR carcinoma*:ti,ab,kw OR 'chronic respiratory disease*':ti,ab,kw OR copd:ti,ab,kw OR 'chronic obstructive pulmonary disease':ti,ab,kw OR emphysema:ti,ab,kw OR 'chronic bronchitis':ti,ab,kw OR asthma:ti,ab,kw OR stroke:ti,ab,kw OR cerebrovascular:ti,ab,kw OR 'chronic kidney disease':ti,ab,kw OR ckd:ti,ab,kw OR renal:ti,ab,kw OR nephropath*:ti,ab,kw OR 'kidney disease*':ti,ab,kw OR 'renal insufficien*':ti,ab,kw OR 'liver disease*':ti,ab,kw OR hepatic:ti,ab,kw OR cirrhosis:ti,ab,kw |
| #3 | #1 OR #2 |
| #4 | 'eHealth literacy':ti,ab,kw OR 'e-health literacy':ti,ab,kw OR ehealth literacy:ti,ab,kw OR 'digital health literacy':ti,ab,kw OR 'digital literacy':ti,ab,kw OR 'computer literacy':ti,ab,kw OR 'internet literacy':ti,ab,kw OR 'telehealth literacy':ti,ab,kw OR 'mhealth literacy':ti,ab,kw OR 'mobile health literacy':ti,ab,kw |
| #5 | interven*:ti,ab,kw OR trial*:ti,ab,kw OR program*:ti,ab,kw OR training:ti,ab,kw OR education:ti,ab,kw OR random*:ti,ab,kw OR 'quasi-experiment*':ti,ab,kw OR controlled:ti,ab,kw |
| #6 | #3 AND #4 AND #5 |

**Supplementary Table 3. Search strategy for Web of Science on mHealth interventions and eHealth literacy in patients with chronic diseases (search date up to February 12, 2026).**

| **Number** | **Search terms** |
| --- | --- |
| #1 | TS=(chronic disease* OR chronic illness* OR noncommunicable disease* OR NCD* OR multimorbid* OR comorbid* OR diabetes OR diabetic* OR "type 2 diabetes" OR T2DM OR "type 1 diabetes" OR hypertension OR hypertens* OR "cardiovascular disease*" OR "heart disease*" OR coronary OR "coronary artery disease" OR myocardial infarct* OR atherosclero* OR "heart failure" OR stroke OR cerebrovascular OR "brain attack" OR "chronic respiratory disease*" OR COPD OR "chronic obstructive pulmonary disease" OR emphysema OR "chronic bronchitis" OR asthma OR neoplasm* OR cancer* OR tumor* OR tumour* OR malignan* OR carcinoma* OR "chronic kidney disease" OR CKD OR renal OR nephropath* OR "kidney disease*" OR "renal insufficien*" OR "liver disease*" OR hepatic OR cirrhosis) |
| #2 | TS=("eHealth literacy" OR "e-health literacy" OR ehealth literacy OR "digital health literacy" OR "digital literacy" OR "computer literacy" OR "internet literacy" OR "telehealth literacy" OR "mhealth literacy" OR "mobile health literacy" OR ("health literacy" NEAR/3 (digital OR electronic OR online OR ehealth OR telehealth OR mhealth))) |
| #3 | TS=(interven* OR trial* OR program* OR training OR education OR random* OR "quasi-experiment*" OR controlled) |
| #4 | #1 AND #2 AND #3 |

**Supplementary Table 4. Search strategy for the Cochrane Library on mHealth interventions and eHealth literacy in patients with chronic diseases (search date up to February 12, 2026).**

| **Number** | **Search terms** |
| --- | --- |
| #1  #2  #3  #4  #5  #6  #7  #8  #9  #10  #11  #12  #13  #14  #15  #16  #17  #18  #19 | MeSH descriptor: [Chronic Disease] explode all trees MeSH descriptor: [Cardiovascular Diseases] explode all trees MeSH descriptor: [Neoplasms] explode all trees MeSH descriptor: [Respiratory Tract Diseases] explode all trees MeSH descriptor: [Pulmonary Disease, Chronic Obstructive] explode all trees MeSH descriptor: [Asthma] explode all trees MeSH descriptor: [Diabetes Mellitus] explode all trees MeSH descriptor: [Hypertension] explode all trees MeSH descriptor: [Stroke] explode all trees MeSH descriptor: [Cerebrovascular Disorders] explode all trees MeSH descriptor: [Kidney Diseases] explode all trees MeSH descriptor: [Renal Insufficiency, Chronic] explode all trees MeSH descriptor: [Liver Diseases] explode all trees (#1 OR #2 OR #3 OR #4 OR #5 OR #6 OR #7 OR #8 OR #9 OR #10 OR #11 OR #12 OR #13) ((chronic NEXT disease*) OR (chronic NEXT illness*) OR (noncommunicable NEXT disease*) OR ncd* OR multimorbid* OR comorbid* OR (cardiovascular NEXT disease*) OR (heart NEXT disease*) OR coronary OR (coronary NEXT artery NEXT disease) OR (myocardial NEXT infarct*) OR atherosclero* OR (heart NEXT failure) OR hypertens* OR diabetes OR diabetic* OR (type NEXT 2 NEXT diabetes) OR (type NEXT 1 NEXT diabetes) OR neoplasm* OR cancer* OR tumor* OR tumour* OR malignan* OR carcinoma* OR (chronic NEXT respiratory NEXT disease*) OR copd OR (chronic NEXT obstructive NEXT pulmonary NEXT disease) OR emphysema OR (chronic NEXT bronchitis) OR asthma OR stroke OR cerebrovascular OR (brain NEXT attack) OR (chronic NEXT kidney NEXT disease) OR ckd OR renal OR nephropath* OR (kidney NEXT disease*) OR (liver NEXT disease*) OR hepatic OR cirrhosis):ti,ab,kw #14 OR #15 (eHealth literacy OR e-health literacy OR ehealth literacy OR digital health literacy OR digital literacy OR computer literacy OR internet literacy OR telehealth literacy OR mhealth literacy OR mobile health literacy):ti,ab,kw (interven* OR trial* OR program* OR training OR education OR random* OR quasi-experiment* OR controlled):ti,ab,kw #16 AND #17 AND #18 |

**Supplementary Table 5. Search strategy for China National Knowledge Infrastructure (CNKI) on mHealth interventions and eHealth literacy in patients with chronic diseases (search date up to February 12, 2026).**

| **Number** | **Search terms** |
| --- | --- |
| #1 | (篇关摘:慢性病 OR 篇关摘:慢性疾病 OR 篇关摘:慢性非传染性疾病 OR 篇关摘:非传染性疾病 OR 篇关摘:NCD OR 篇关摘:多病共存 OR 篇关摘:共病 OR 篇关摘:心血管疾病 OR 篇关摘:冠心病 OR 篇关摘:冠状动脉疾病 OR 篇关摘:心脏病 OR 篇关摘:心力衰竭 OR 篇关摘:糖尿病 OR 篇关摘:2型糖尿病 OR 篇关摘:高血压 OR 篇关摘:脑卒中 OR 篇关摘:脑梗死 OR 篇关摘:脑出血 OR 篇关摘:慢阻肺 OR 篇关摘:慢性阻塞性肺疾病 OR 篇关摘:COPD OR 篇关摘:哮喘 OR 篇关摘:肿瘤 OR 篇关摘:癌 OR 篇关摘:癌症 OR 篇关摘:恶性肿瘤 OR 篇关摘:慢性肾病 OR 篇关摘:肾功能不全 OR 篇关摘:肾病 OR 篇关摘:慢性肝病 OR 篇关摘:肝硬化) |
| #2 | (篇关摘:电子健康素养 OR 篇关摘:电子健康素养能力 OR 篇关摘:数字健康素养 OR 篇关摘:数字素养 OR 篇关摘:计算机素养 OR 篇关摘:网络素养) |
| #3 | (篇关摘:干预 OR 篇关摘:试验 OR 篇关摘:随机 OR 篇关摘:对照 OR 篇关摘:培训 OR 篇关摘:教育 OR 篇关摘:项目 OR 篇关摘:影响) |
| #4 | #1 AND #2 AND #3 |

**Supplementary Table 6. Search strategy for the Wanfang Database on mHealth interventions and eHealth literacy in patients with chronic diseases (search date up to February 12, 2026).**

| **Number** | **Search strategy** |
| --- | --- |
| #1 | (题名或关键词=(慢性病 OR 慢性疾病 OR 慢性非传染性疾病 OR 非传染性疾病 OR NCD OR 多病共存 OR 共病 OR 心血管疾病 OR 冠心病 OR 冠状动脉疾病 OR 心脏病 OR 心力衰竭 OR 糖尿病 OR 2型糖尿病 OR 高血压 OR 脑卒中 OR 脑梗死 OR 脑出血 OR 慢阻肺 OR 慢性阻塞性肺疾病 OR COPD OR 哮喘 OR 肿瘤 OR 癌 OR 癌症 OR 恶性肿瘤 OR 慢性肾病 OR 肾功能不全 OR 肾病 OR 慢性肝病 OR 肝硬化)) |
| #2 | (题名或关键词=(电子健康素养 OR 数字健康素养 OR 数字素养 OR 计算机素养 OR 网络素养)) |
| #3 | (题名或关键词=(干预 OR 试验 OR 随机 OR 对照 OR 培训 OR 教育 OR 项目 OR 影响)) |
| #4 | #1 AND #2 AND #3 |

**Supplementary Table 7. Search strategy for the Chinese Biomedical Literature Database (CBM) on mHealth interventions and eHealth literacy in patients with chronic diseases (search date up to February 12, 2026).**

| **Number** | **Search terms** |
| --- | --- |
| #1 | ("慢性病"[不加权:扩展] OR "慢性疾病"[不加权:扩展] OR "慢性非传染性疾病"[不加权:扩展] OR "非传染性疾病"[不加权:扩展] OR "多病共存"[不加权:扩展] OR "共病"[不加权:扩展] OR "心血管疾病"[不加权:扩展] OR "冠心病"[不加权:扩展] OR "心力衰竭"[不加权:扩展] OR "糖尿病"[不加权:扩展] OR "2型糖尿病"[不加权:扩展] OR "高血压"[不加权:扩展] OR "脑卒中"[不加权:扩展] OR "慢性阻塞性肺疾病"[不加权:扩展] OR "哮喘"[不加权:扩展] OR "肿瘤"[不加权:扩展] OR "癌症"[不加权:扩展] OR "恶性肿瘤"[不加权:扩展] OR "慢性肾病"[不加权:扩展] OR "肾功能不全"[不加权:扩展] OR "肾病"[不加权:扩展] OR "慢性肝病"[不加权:扩展] OR "肝硬化"[不加权:扩展]) |
| #2 | "慢性病"[常用字段:智能] OR "慢性疾病"[常用字段:智能] OR "慢性非传染性疾病"[常用字段:智能] OR "非传染性疾病"[常用字段:智能] OR "多病共存"[常用字段:智能] OR "共病"[常用字段:智能] OR "心血管疾病"[常用字段:智能] OR "冠心病"[常用字段:智能] OR "心力衰竭"[常用字段:智能] OR "糖尿病"[常用字段:智能] OR "2型糖尿病"[常用字段:智能] OR "高血压"[常用字段:智能] OR "脑卒中"[常用字段:智能] OR "慢性阻塞性肺疾病"[常用字段:智能] OR "哮喘"[常用字段:智能] OR "肿瘤"[常用字段:智能] OR "癌症"[常用字段:智能] OR "恶性肿瘤"[常用字段:智能] OR "慢性肾病"[常用字段:智能] OR "肾功能不全"[常用字段:智能] OR "肾病"[常用字段:智能] OR "慢性肝病"[常用字段:智能] OR "肝硬化"[常用字段:智能] |
| #3 | #1 OR #2 |
| #4 | "电子健康素养"[常用字段:智能] OR "数字健康素养"[常用字段:智能] OR "数字素养"[常用字段:智能] OR "计算机素养"[常用字段:智能] OR "网络素养"[常用字段:智能] |
| #5 | "干预"[常用字段:智能] OR "试验"[常用字段:智能] OR "随机"[常用字段:智能] OR "对照"[常用字段:智能] OR "培训"[常用字段:智能] OR "教育"[常用字段:智能] OR "项目"[常用字段:智能] OR "影响"[常用字段:智能] |
| #6 | #3 AND #4 AND #5 |

**Supplementary Table 8. Search strategy for the Chinese Science and Technology Periodical Database (VIP) on mHealth interventions and eHealth literacy in patients with chronic diseases (search date up to February 12, 2026).**

| **Number** | **Search terms** |
| --- | --- |
| #1 | 题名或关键词=(慢性病 OR 慢性疾病 OR 慢性非传染性疾病 OR 非传染性疾病 OR NCD OR 多病共存 OR 共病 OR 心血管疾病 OR 冠心病 OR 冠状动脉疾病 OR 心脏病 OR 心力衰竭 OR 糖尿病 OR 2型糖尿病 OR 高血压 OR 脑卒中 OR 脑梗死 OR 脑出血 OR 慢阻肺 OR 慢性阻塞性肺疾病 OR COPD OR 哮喘 OR 肿瘤 OR 癌 OR 癌症 OR 恶性肿瘤 OR 慢性肾病 OR 肾功能不全 OR 肾病 OR 慢性肝病 OR 肝硬化) |
| #2 | 题名或关键词=(电子健康素养 OR 数字健康素养 OR 数字素养 OR 计算机素养 OR 网络素养) |
| #3 | 题名或关键词=(干预 OR 试验 OR 随机 OR 对照 OR 培训 OR 教育 OR 项目 OR 影响) |
| #4 | #1 AND #2 AND #3 |

**Research results**

We have re-conducted searches in databases including PubMed, Web of Science, Embase, Cochrane Library, WanFang, CNKI, CQVIP, and CBM, adhering to the original search strategy. The search period was from from inception to February 2026. A total of 4057 publications were identified. The remaining four publications were excluded after full-text review, as they were deemed ineligible for inclusion in this study. Detailed search results are as follows:

**1.Search strategy of PubMed**


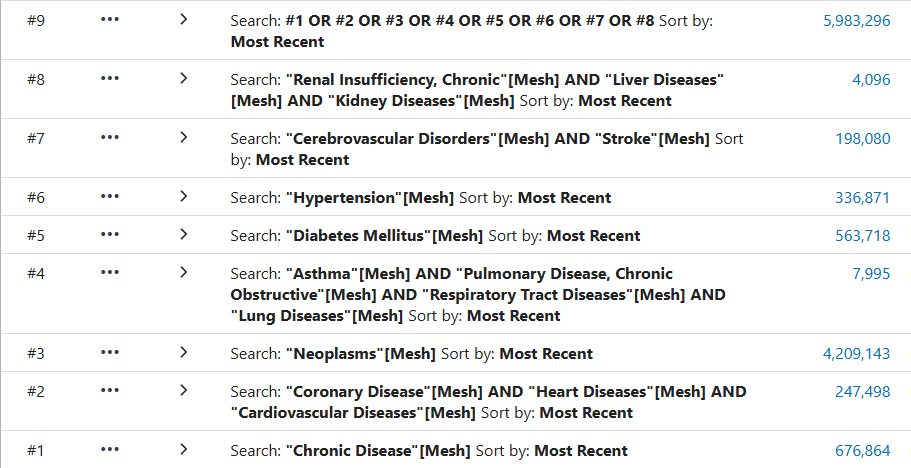

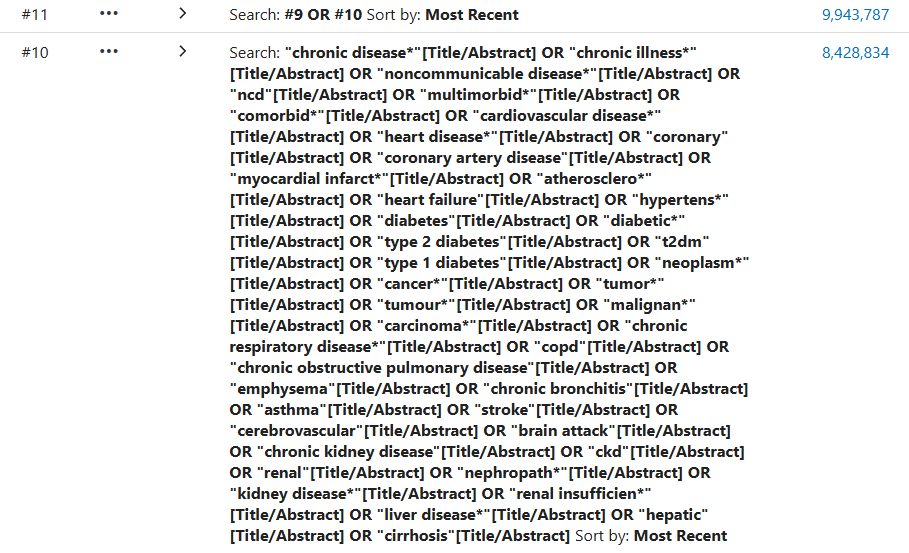

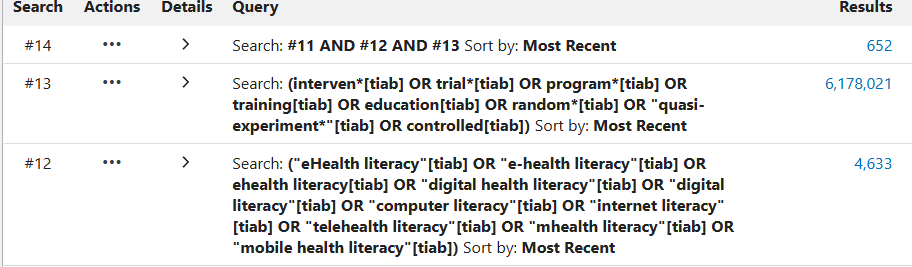


**2.Search strategy for Embase**


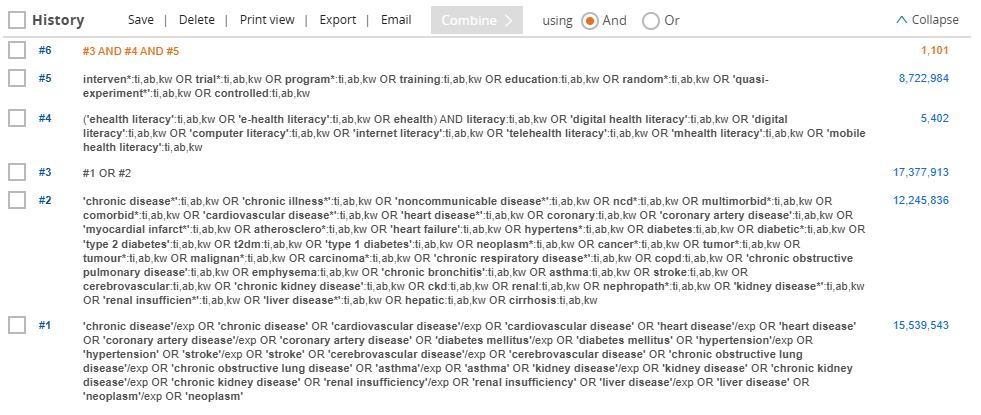


**3. Search strategy for Web of Science**


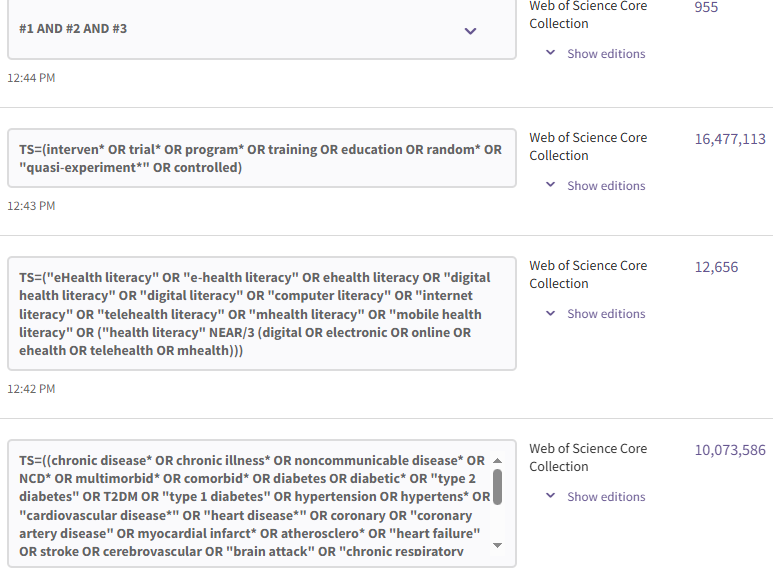


**4. Search strategy for the Cochrane Library**


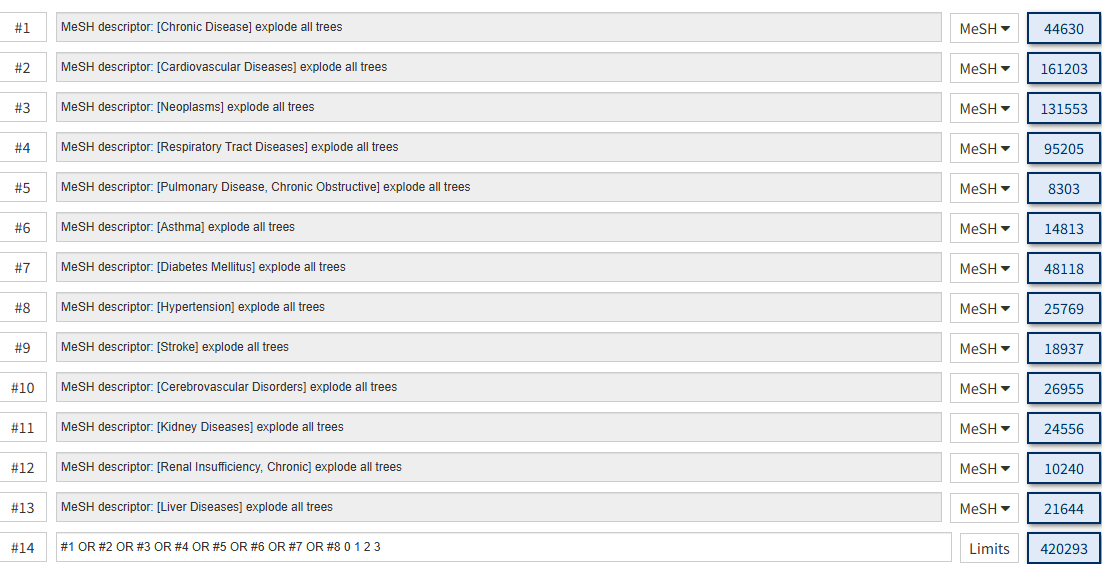

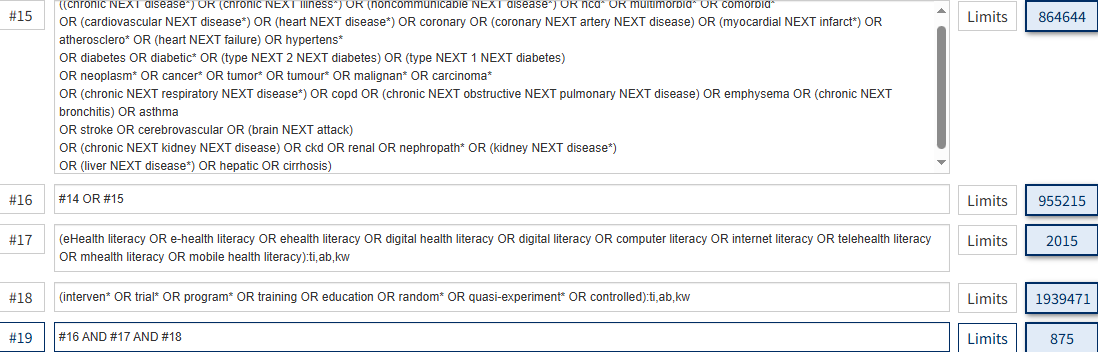


**5. Search strategy for the CNKI**


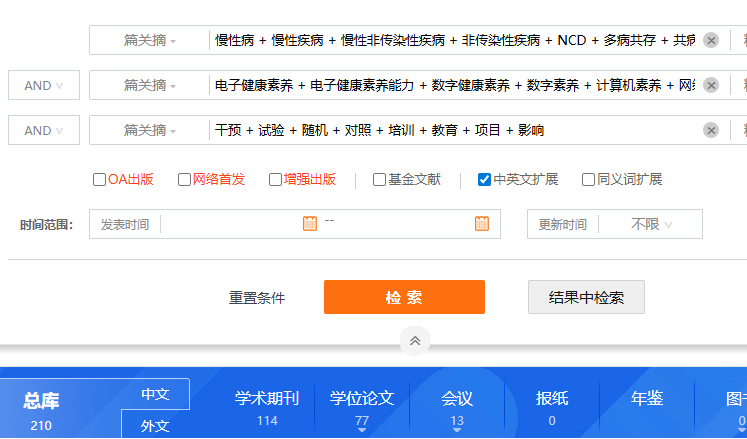


**6. Search strategy for the Wanfang Database**


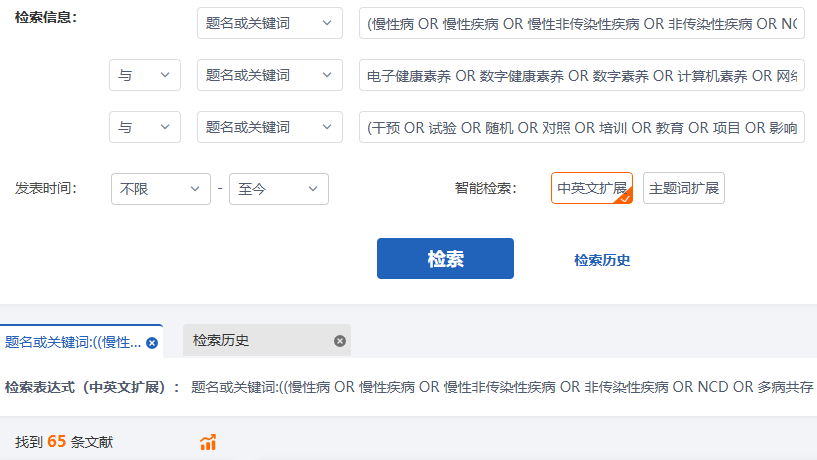


7. **Search strategy for the Chinese Biomedical Literature Database (CBM)**


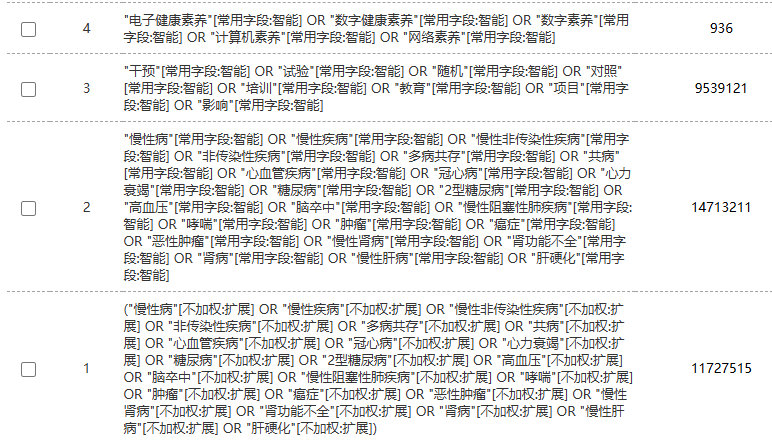

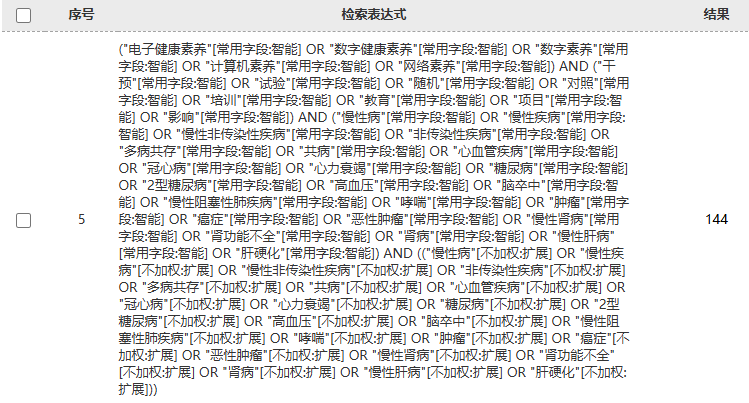


**8.** **Search strategy for the Chinese Science and Technology Periodical Database (VIP)**


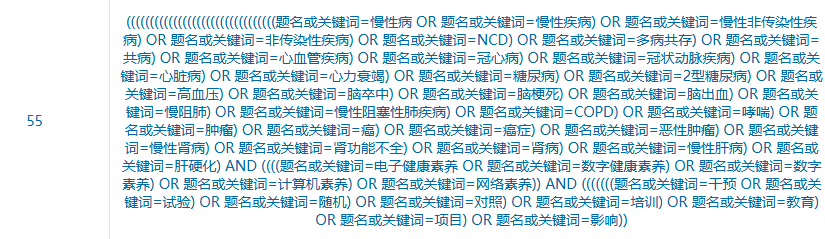


**Supplementary Table 9. Egger's Test for Small-Study Effects in Quasi-Experimental Studies (n = 5)**

| Term | Value |
| --- | --- |
| β₁ | 1.57 |
| SE | 1.910 |
| z | 0.82 |
| *P* | 0.4105 |

**Note.** The regression-based Egger’s test was performed under a random-effects model (Hartung–Knapp–Sidik–Jonkman). H₀: β₁ = 0 (no small-study effects). The non-significant P-value suggests no evidence of small-study effects was detected in the present set of studies. However, it is important to note that the statistical power of Egger’s test is limited when the number of included studies is small (k=5 in this analysis). Therefore, this result should be interpreted with caution, and the possibility of publication bias cannot be definitively ruled out.

**Supplementary Table 10. Summary of Implementation Characteristics of Mobile Health Interventions (n=15)**

| **Number** | **Author** | **Target**  **Population** | **Intervention**  **Site** | **Intervention Methods** | **Platform**  **/Tools** | **Frequency** | **Session Duration** |
| --- | --- | --- | --- | --- | --- | --- | --- |
| 1 | Yu | Young & Middle-Aged Stroke Patients | Hospital | Mixed Model (Online +face to face) | Online: WeChat, DingTalk (later switched to WeChat) for communication & task assignment.  face to face: eHealth literacy intervention manual (online + printed). | Twice a week | 20–30 minutes |
| 2 | Nahm et al | Chronic disease patients | Community | Online-only model | Online course constructed using the Blackboard e-learning platform. | One module per week (self-paced) | Not fixed |
| 3 | Hu | Elderly chronic disease patients | Hospital | Mixed Model (Online +face to face) | Online: Conducted via the hospital's "317 Nursing" platform (a nursing health education platform).  face to face: Researchers demonstrated using unified tablets; patients practiced using their own mobile phones. | Once daily during hospitalization | 25–35 minutes |
| 4 | Jiang et al | Hypertension patients | Community | Primarily online | Participants used a mini-program independently to record blood pressure and read articles; researchers monitored data via a backend system. | Push notifications weekly/fortnightly | Not fixed |

**Continuation of Supplementary Table 10. Summary of Implementation Characteristics of Mobile Health Interventions (n=15)**

|  |  | **Target** | **Intervention** | **Intervention Methods** | **Platform** | **Frequency** | **Session Duration** |
| --- | --- | --- | --- | --- | --- | --- | --- |
| **Number** | **Author** | **Population** | **Site** |  | **/Tools** |  |  |
| 5 | Guo et al | Diabetes patients | Hospital | Mixed Model (Online +face to face) | Online: Interactive multimedia modules created with iBook Author software. | One-time education session | 30–60 minutes |
|  |  |  |  |  | face to face: Educational content delivered via iPad. |  |  |
| 6 | Parker et al | Chronic disease patients | Community | Mixed Model (Online +face to face) | Mobile application "mysnapp" | Not mentioned | |
| 7 | Chiu et al | Chronic disease patients | Community | face to face | Paper-based manual | Once per week | 90 minutes per session |
| 8 | Redfern et al | Cardiovascular disease patients | Community | Online | "CONNECT"web-based application / consumer app | Not prescribed, patient self-managed/accessed | |
| 9 | Kastner et al | Chronic disease patients | Community | Online | "KeepWell" responsive web application | Not prescribed, patient self-managed/accessed | |
| 10 | Melholt et al | Heart disease patients | Hospital | Online | "Active Heart" web portal | Not prescribed, patient self-managed/accessed | |

**Continuation of Supplementary Table 10. Summary of Implementation Characteristics of Mobile Health Interventions (n=15)**

|  |  | **Target** | **Intervention** | **Intervention Methods** | **Platform** | **Frequency** | **Session Duration** |
| --- | --- | --- | --- | --- | --- | --- | --- |
| **Number** | **Author** | **Population** | **Site** |  | **/Tools** |  |  |
| 11 | Cheng et al | Type 2 diabetes patients | Community | online + face-to-face | Wearable devices , health apps | 6 biweekly sessions over 3 months | Approx. 90 min/session |
| 12 | Son et al | Heart failure patients | Hospital | face to face | Hospital-developed mobile app | Monthly sessions for 3 months | 30 min/session |
| 13 | Spindler et al | Heart failure patients | Hospital | Online | self-tracking devices | 12 months | continuous use |
| 14 | Gao et al | Older adults (≥65 years) with Chronic Obstructive Pulmonary Disease | Hospital | Online + Face-to-face | Online: WeChat (communication, Q&A), “PeR” public account (course videos), “PeR” platform (tele-PR delivery, progress tracking). Face-to-face: Printed course manuals, reflective journals. | 8-week | Not fixed (self-paced). Tele-PR exercise: 20-30 min/session. |
| 15 | Lyles et al | patients with chronic diseases | Community | Online + Face-to-face | Online: LearnerWeb platform (hosted training videos), MYSFHEALTH patient portal. Face-to-face: Informational pamphlet, handout with access instructions. | One-time training session. Portal use assessed at 3-6 months. | Not fixed (self-paced). |

**Supplementary Table 11. Data extraction table for meta-analysis, including baseline and post-intervention eHealth literacy scores (N=11)**

|  |  |  | **Baseline** | | | | | | | **post-intervention** | | | | |  |  |
| --- | --- | --- | --- | --- | --- | --- | --- | --- | --- | --- | --- | --- | --- | --- | --- | --- |
|  |  |  | **Experimental group** | | | **Control group** | | | **Experimental**  **group** | | | **Control group** | | | |  |
| Number | **Study(year) Study design** | **Author** | M | SD | Total | | M | SD | Total | M | SD | Total | M | SD | | Total |
| 1 | （2022 RCT | Yu | 28.86 | 2.68 | 29 | | 27.72 | 3.68 | 29 | 36.79 | 1.11 | 29 | 31.9 | 2.08 | | 29 |
| 2 | （2019）RCT | Nahm et al | 30.62 | 5.02 | 138 | | 28.86 | 5.26 | 134 | 31.58 | 5.21 | 113 | 28.44 | 6.16 | | 127 |
| 3 | （2024）QED | Hu | 14 | 1.345 | 38 | | 15 | 1.29 | 35 | 24 | 12.1 | 38 | 15 | 11.62 | | 35 |
| 4 | （2024）QED | Jiang et al | 25.57 | 5.97 | 30 | | 25.43 | 6.25 | 30 | 29.27 | 4.23 | 30 | 26.67 | 4.92 | | 30 |
| 5 | （2023）QED | Guo et al | 30.65 | 4.859 | 96 | | 31.806 | 5.6 | 36 | 31.72 | 4.287 | 96 | 32.222 | 4.486 | | 36 |
| 6 | （2022）RCT | Parker et al | 22.5 | 5.3 | 69 | | 23.8 | 5.2 | 41 | 29.5 | 4.7 | 25 | 26.5 | 6.2 | | 27 |
| 7 | (2016) QED | Chiu et al | 30.9 | 5.1 | 39 | |  |  |  | 31.7 | 5.4 | 20 |  |  | |  |
| 8 | (2024) RCT | Cheng et al | 90.96 | 19.0 | 46 | | NA | NA | NA | 96.67 | 18.0 | 46 | NA | NA | | NA |

**Continuation of Supplementary Table 11. Data extraction table for meta-analysis, including baseline and post-intervention eHealth literacy scores(n=11)**

| **Number** | **Study(year) Study design** | **Author** | **M** | **SD** | **Total** | **M** | **SD** | **Total** | **M** | **SD** | **Total** | **M** | **SD** | **Total** |
| --- | --- | --- | --- | --- | --- | --- | --- | --- | --- | --- | --- | --- | --- | --- |
| 9 | (2022) QED | Son et al | 24.32 | 9.55 | 50 | 25.58 | 9.79 | 50 | 28.10 | 9.16 | 50 | 27.20 | 8.73 | 50 |
| 10 | (2026) RCT | Gao et al | 23.49 | 5.51 | 65 | 24.68 | 5.73 | 65 | 32.97 | 4.44 | 65 | 29.48 | 5.14 | 65 |
| 11 | (2019) RCT | Lyles et al | 14.4 | 3.7 | 75 | NA | NA | NA | 16.2 | 2.4 | 75 | NA | NA | NA |

**Note:** RCT: randomized controlled trail; QED: quasi-experiment design; NA: data not available in the original study.
