# Supplementary material for: Effectiveness of mHealth Interventions for Improving eHealth Literacy Among Patients With Chronic Diseases: Meta-Analysis and Systematic Review
Source: J Med Internet Res. 2026 Apr 17;28:e82004. doi: 10.2196/82004 (PMC13089675; doi:10.2196/82004)
Supplement: Multimedia Appendix 3 [file jmir-v28-e82004-s003.pdf]

GRADE for evidence

Outcomes	Subgroup	Study characteristic	Absolute effect (SMD)	Risk of bias	Indirectness	Inconsistency	Imprecision	Other consideration	Rating
Randomized Controlled Trials									
eHealth literacy	Overall	681 patients from 6 studies	1.19 (0.14 to 2.23)	Not serious[a]	Not serious	Serious[b]	Not serious[c]	Not serious[d]	Moderate
eHealth literacy	Specific disease target	4 studies	1.61 (0.16 to 3.06)	Not serious[a]	Not serious	Serious[b]	Serious[e]	Not serious	Low
eHealth literacy	General chronic disease target	2 studies	0.36 (0.00 to 0.73)	Not serious[a]	Not serious	Not serious[f]	Serious [g]	Not serious	Moderate
eHealth literacy	Intervention duration ≥3 months	3 studies	1.79 (-0.22 to 3.80)	Not serious[a]	Not serious	Serious[h]	Not serious	Not serious	Moderate
eHealth literacy	Intervention duration <3 months	3 studies	0.61 (0.09 to 1.13)	Not serious[a]	Not serious	Serious[i]	Serious [j]	Not serious	Low
Quasi-experimental studies									
eHealth literacy	Overall	413 patients from 5 studies	0.51 (0.15 to 0.87)	Serious[k]	Not serious	Serious[l]	Not serious[m]	Not serious	Low

Footnotes and Explanations:

a. Risk of bias: Not serious. Although some studies had limitations in blinding and implementation (see Manuscript Figure 2a), the overall risk of bias was acceptable and did not warrant downgrading.
b. Inconsistency: Serious. Pooled results showed very high heterogeneity (I^2 = 97.75%), not fully explained by subgroup analyses; the prediction interval was extremely wide (overall PI -2.68 to 5.05), suggesting substantial variability in true effects across settings.
c. Imprecision: Not serious. The 95% confidence interval of the pooled effect did not cross the null, indicating statistical significance.
d. Other consideration: Publication bias:Not serious. Egger's test was not statistically significant (P = 0.2245).
e. Imprecision: Serious. The confidence interval was wide (1.61, 95% CI 0.16–3.06), and the prediction interval was very wide (-5.40 to 8.63), indicating poor precision.
f. Inconsistency: Not serious. Heterogeneity within this subgroup was moderate (I^2 = 68.46%), considered acceptable.
g. Imprecision: Serious. The confidence interval included the null (0.36, 95% CI 0.00–0.73), suggesting the effect may not be clinically meaningful.
h. Inconsistency: Serious. Heterogeneity was high (I^2 = 88.04%) and the prediction interval wide (-5.72 to 6.95).
i. Inconsistency: Serious. Heterogeneity was very high (I^2 = 97.52%) and the prediction interval extremely wide (-23.98 to 27.56).
j. Imprecision: Serious. The confidence interval crossed the null (1.79, 95% CI -0.22–3.80), indicating non-significance.
k. Risk of bias: Serious. Based on ROBINS-I assessment, the quasi-experimental studies had serious confounding bias (see Manuscript Figure 2b), inherent to their design.
l. Inconsistency: Serious. Pooled results showed moderate to high heterogeneity (I^2 = 71.63%) and a wide prediction interval (-0.73 to 1.75).
m. Imprecision: Not serious. The 95% confidence interval did not cross the null (0.51, 95% CI 0.15–0.87), indicating statistical significance.
