# Supplementary material for: Effectiveness of mHealth Interventions for Improving eHealth Literacy Among Patients With Chronic Diseases: Meta-Analysis and Systematic Review
Source: J Med Internet Res. 2026 Apr 17;28:e82004. doi: 10.2196/82004 (PMC13089675; doi:10.2196/82004)
Supplement: Checklist 1 [file jmir-v28-e82004-s004.pdf]

Section/topic	#	Checklist item	Location(s) Reported
**INFORMATION SOURCES AND METHODS**			
Database name	1	Name each individual database searched, stating the platform for each.	P6
Multi-database searching	2	If databases were searched simultaneously on a single platform, state the name of the platform, listing all of the databases searched.	P6
Study registries	3	List any study registries searched.	P6
Online resources and browsing	4	Describe any online or print source purposefully searched or browsed (e.g., tables of contents, print conference proceedings, web sites), and how this was done.	P6
Citation searching	5	Indicate whether cited references or citing references were examined, and describe any methods used for locating cited/citing references (e.g., browsing reference lists, using a citation index, setting up email alerts for references citing included studies).	P6
Contacts	6	Indicate whether additional studies or data were sought by contacting authors, experts, manufacturers, or others.	P7
Other methods	7	Describe any additional information sources or search methods used.	P7
**SEARCH STRATEGIES**			
Full search strategies	8	Include the search strategies for each database and information source, copied and pasted exactly as run.	Supplement aryTable S1 –S8
Limits and restrictions	9	Specify that no limits were used, or describe any limits or restrictions applied to a search (e.g., date or time period, language, study design) and provide justification for their use.	P7
Search filters	10	Indicate whether published search filters were used (as originally designed or modified), and if so, cite the filter(s) used.	P7
Prior work	11	Indicate when search strategies from other literature reviews were adapted or reused for a substantive part or all of the search, citing the previous review(s) .	P7
Updates	12	Report the methods used to update the search(es) (e.g., rerunning searches, email alerts).	P7
Dates of searches	13	For each search strategy, provide the date when the last search occurred.	Suppleme ntary data Table S1–S8 Title
**PEER REVIEW**			
Peer review	14	Describe any search peer review process.	P7
**MANAGING RECORDS**			
Total Records	15	Document the total number of records identified from each database and other information sources.	P12
Deduplication	16	Describe the processes and any software used to deduplicate records from multiple database searches and other information sources.	P12

PRISMA-S: An Extension to the PRISMA Statement for Reporting Literature Searches in Systematic Reviews
Rethlefsen ML, Kirtley S, Waffenschmidt S, Ayala AP, Moher D, Page MJ, Koffel JB, PRISMA-S Group.
Last updated February 27, 2020.
